# People With High Autistic Traits Show Fewer Consensual Crossmodal Correspondences Between Visual Features and Tastes

**DOI:** 10.3389/fpsyg.2021.714277

**Published:** 2021-09-08

**Authors:** Na Chen, Katsumi Watanabe, Makoto Wada

**Affiliations:** ^1^Department of Rehabilitation for Brain Functions, Research Institute of National Rehabilitation Center for Persons With Disabilities, Tokorozawa, Japan; ^2^Faculty of Science and Engineering, Waseda University, Tokyo, Japan; ^3^Faculty of Arts, Design, and Architecture, University of New South Wales, Sydney, NSW, Australia

**Keywords:** color–taste association, shape–taste association, shape–color association, autistic traits, crossmodal correspondence

## Abstract

Crossmodal correspondences between visual features (e.g., color/shape) and tastes have been extensively documented in recent years. Visual colors and shapes have been shown to consensually match to specific tastes. Meanwhile, individuals with autism spectrum disorder are reported to have atypical sensory processing and deficits in multisensory integration. However, the influence of autistic traits on the formation of such correspondences is relatively unknown. Here, we examined whether autistic traits could influence visual–taste associations using an online questionnaire survey among Japanese participants. The results showed that the participants exhibited strong color–taste, shape–taste, and shape–color associations, and the proportions of choosing the consensual color–taste/shape–color associations were significantly associated with autistic traits. The participants with higher autistic quotient scores chose fewer of the consensual color–taste/shape–color associations while there was no difference in choosing shape–taste associations. We interpreted the results as statistical learning with a reduced prior knowledge effect in participants with higher autistic quotient scores.

## Introduction

Which taste best matches the color yellow? When asked to match a basic taste with a color, people choose some tastes more frequently than the others (e.g., yellow–sour/red–sweet; O’Mahony, 1983; Spence et al., 2015; Woods and Spence, 2016; Velasco et al., 2016a). These naturally biased color–taste associations might be explained by learning with frequent exposure to the color of foods/drinks in the environment (Spence et al., 2015; Saluja and Stevenson, 2018; Higgins and Hayes, 2019; Spence, 2019). Spence (2011) defined the tendency to match distinct features or dimensions of experience across sensory modalities as crossmodal correspondences. There are four main types of crossmodal correspondences: structural, statistical, emotional, and semantic. Structural correspondence arises from the peculiarities of the neural systems when the sensory information is coded; for example, some correspondences arise when two unimodal stimulus properties are represented by the same neural substrate or processed in neighboring or interconnected brain areas (e.g., brightness–loudness; Lewkowicz and Turkewitz, 1980; Ramachandran and Hubbard, 2001; Walsh, 2003; Mondloch and Maurer, 2004; Rouw and Scholte, 2007; Knöferle and Spence, 2012). In addition, researchers reported that some correspondences are innate (e.g., brightness–auditory pitch; Ludwig et al., 2011), though other researchers argued that the correspondence could be acquired by correlations in the environment (Spence and Deroy, 2012a). Statistical correspondence is acquired through statistical learning with repeated exposures to co-occurrences in the natural environment (a Bayesian prior, e.g., lightness–position; Parise and Spence, 2013; Parise et al., 2014; Brunel et al., 2015). Emotional correspondence arises based on the emotional associations that people have with sensory stimuli (e.g., the valence or arousal; music–color; Palmer et al., 2013; Wang et al., 2016; Turoman et al., 2018; Aryani et al., 2020; Motoki et al., 2020; Spence, 2020). Semantic correspondence stems from the linguistic terms or semantic information underlying these sensory properties (e.g., “high/low” pitch in sound–position; Melara and Marks, 1990; Chen et al., 2015a; Velasco et al., 2016b). These accounts of crossmodal correspondence are intertwined and not mutually exclusive and some crossmodal correspondences could be explained by combinations of more than one hypothesis (Spence, 2011).

Crossmodal correspondences between visual features (color/shape) and basic tastes have been widely studied, and specific color–taste/shape–taste associations have been observed (e.g., yellow–sour, red–sweet, angular shapes–sour/bitter, and round shapes–sweet associations; Spence and Gallace, 2011; Spence, 2012, 2019; Spence and Ngo, 2012; Bremner et al., 2013; Wan et al., 2014; Spence et al., 2015; Velasco et al., 2015, 2016a,b; Saluja and Stevenson, 2018; Turoman et al., 2018; Higgins and Hayes, 2019; Spence and Levitan, 2021). Statistical/semantic/emotional correspondence hypotheses were suggested to explain some of these visual–taste associations. For example, color–taste associations might be explained by the statistical learning of co-occurrences in the environment (e.g., colors of foods and drinks; Spence et al., 2015; Foroni et al., 2016; Saluja and Stevenson, 2018; Higgins and Hayes, 2019; Spence, 2019). Meanwhile, shape–taste associations might be based on the semantic/emotional correspondence account, such as the hedonic dimension (Spence and Deroy, 2012b, 2013a; Salgado-Montejo et al., 2015; Velasco et al., 2015, 2016b; Blazhenkova and Kumar, 2018; Turoman et al., 2018; Motoki and Velasco, 2021). In addition, Wan et al. (2014) examined visual (color/shape/texture)–taste associations among participants from four different cultures (i.e., China, India, Malaysia, and the United States). They found that some visual (color/shape)–taste associations are immune to cultural backgrounds, and some are culturally specific, implying the different natures of visual–taste associations. Simultaneously, studies have shown that people also systematically associate geometric shapes with colors (e.g., circle–red and triangle–yellow), which might be explained by statistical/emotional/semantic correspondence hypotheses (Albertazzi et al., 2013; Malfatti, 2014; Chen et al., 2015a, 2016; Dreksler and Spence, 2019; Hanada, 2019). Possibly, people learned to establish crossmodal correspondences between colors, shapes, and tastes and exploit that mapping information (i.e., a Bayesian prior) to integrate multisensory features efficiently (Spence, 2011; Parise and Spence, 2013; Spence and Deroy, 2013b; Chen et al., 2015b; Chen and Watanabe, 2021).

Autism spectrum disorder (ASD) refers to a type of neurodevelopmental disorder, characterized by behavioral dysfunctions in specific developmental areas, such as social interaction, communication, and a restricted range of interests and stereotyped behaviors (American Psychiatric Association, 2013). In addition to social and behavioral symptoms, people with ASD are reported to have difficulties in sensory processing, including over- or under-sensitivity to sensory stimuli, and deficits in multisensory integration (Iarocci and McDonald, 2006; Bennetto et al., 2007; Oberman and Ramachandran, 2008; Foss-Feig et al., 2010; Cascio et al., 2012a; Tavassoli and Baron-Cohen, 2012; Occelli et al., 2013; Stevenson et al., 2014a; Wallace and Stevenson, 2014; DuBois et al., 2017). The impaired ability to integrate multisensory information in individuals with ASD may arise from an underlying impairment in perceiving the relationships between cross-modal inputs (Stevenson et al., 2014a). For example, the most studied “bouba–kiki” effect (i.e., people associate rounded shapes with words like “bouba” or “maluma,” and spiky shapes with words like “kiki” or “takete”; Köhler, 1947) was reported to be less pronounced in individuals with autism compared to controls (Oberman and Ramachandran, 2008; Occelli et al., 2013; Gold and Segal, 2017; Król and Ferenc, 2020). Oberman and Ramachandran (2008) found that children with ASD do not show the “bouba–kiki” effect that neurologically typical adults and children show (the neurotypical children chose expected sound–shape associations 88% of the time, while children with ASD chose only 56% of the time, compared to the chance of 50%). They suggested that an impairment in multisensory integration systems, such as a mirror neuron-like system, prevents the systematic bias for sound–shape mappings in individuals with autism (Iarocci and McDonald, 2006; Rizzolatti et al., 2009). Sensory irregularity in autism may hinder the construction of structural correspondences (Ramachandran and Hubbard, 2001). While individuals with autism were suggested to exhibit impaired ability to integrate multisensory signals, some showed intact or excess integration of low-level multisensory information (e.g., audio–visual integration; Mottron and Burack, 2001; Foss-Feig et al., 2010; Kwakye et al., 2011; Stevenson et al., 2014b). A Bayesian prior framework posits that, individuals with autism perceive the world as a consequence of hypo-priors or reduced reliance on previous perceptual experiences, and that the current sensory input is given proportionately greater weight than the learned priors, resulting in prediction errors, which may be related to atypical multisensory integration (Mitchell and Ropar, 2004; Fiser et al., 2010; Pellicano and Burr, 2012; Van Boxtel and Lu, 2013; Robertson and Baron-Cohen, 2017; Stevenson et al., 2017). Some visual–taste associations are suggested to be statistically constructed; it is possible that the autistic traits interfere with the statistical learning experience and consequently, influence those crossmodal correspondences.

Studies suggested that individuals with ASD, especially in children with ASD, exhibited more restricted food preferences than the typically developed controls, which may be related to atypical sensory processing of visual (color/shape), taste, smell, and/or texture sensory information in autistic perception (Shore, 2001; Lane et al., 2014; Zobel-Lachiusa et al., 2015; Chistol et al., 2018). To our knowledge, there is almost no study that examined crossmodal correspondences between taste perception and other sensory dimensions in individuals with ASD. Learning the influence of autistic traits on crossmodal correspondence between taste and visual (color/shape) may help to shed light on the eating problems in ASD. In the present study, we aimed to examine the relationship between visual (color–shape)–taste associations and autistic tendency to reveal the effect of autistic traits on these associations. It has been suggested that ASD characteristics are continuously distributed in the general population (Baron-Cohen et al., 2001). The Autism-Spectrum Quotient (AQ) has been developed as a measure to assess autistic traits among typical-intelligence individuals both with and without ASD diagnoses (Baron-Cohen et al., 2001; Wakabayashi et al., 2004, 2006). Here, an online questionnaire survey with a short version of the AQ questionnaire (AQ-10) was used (Kurita et al., 2005; Booth et al., 2013; Maeda et al., 2017). We predicted that participants would show specific visual(color–shape)–taste associations, which were correlated with the autistic tendency, and the participants with higher AQ scores show fewer of the consensual (i.e., commonly agreed) visual–taste associations. Furthermore, we used the same visual color and shape stimuli and the forced choice method from a previous study, with the aim to compare visual(color/shape)–taste associations in Japanese and that from the other four cultural backgrounds (i.e., China, India, Malaysia, and the United States; Wan et al., 2014).

## Materials and Methods

### Participants

Eighty-five Japanese (52 females; 57 participants were 15–29years old, 20 participants were 30–49years old, and eight participants were above 50years of age) volunteered to participate in an online questionnaire survey. Sample size was determined by the criteria that are needed to be able to detect a correlation coefficient of 0.3 with an alpha of 0.05 and power of 80% (Bujang and Baharum, 2016). The participants were mainly from research participant pools of the National Rehabilitation Center for Persons with Disabilities and students of Waseda University. Eleven participants reported being diagnosed with developmental disorders (four participants with ASD, one participant with ADHD, one participant with intellectual disability, one participant with learning disability, two participants with depression, one participant with panic disorder, and one participant with cyclic vomiting syndrome). Three participants who filled out the questionnaire with missing responses were excluded, and 82 participants’ responses were used for data analysis. All participants were Japanese and agreed to participate in the questionnaire survey. This study was reviewed and approved by the Ethics Committee of the National Rehabilitation Center for Persons with Disabilities (2020-082).

### Materials

This study was conducted using Google forms (see Supplementary Material for details).^1^ The questionnaire was presented in Japanese. Text descriptors of “甘味(sweet),” “酸味(sour),” “旨味(umami),” “苦味(bitter),” and “塩味(salty)” were used as the five basic taste terms, presented in Japanese MS Gothic font size 9. The text description and the image were presented with a white background, and the questionnaire survey was designed with a gray background (RGB: 240, 240, 240).

**Figure 1:**
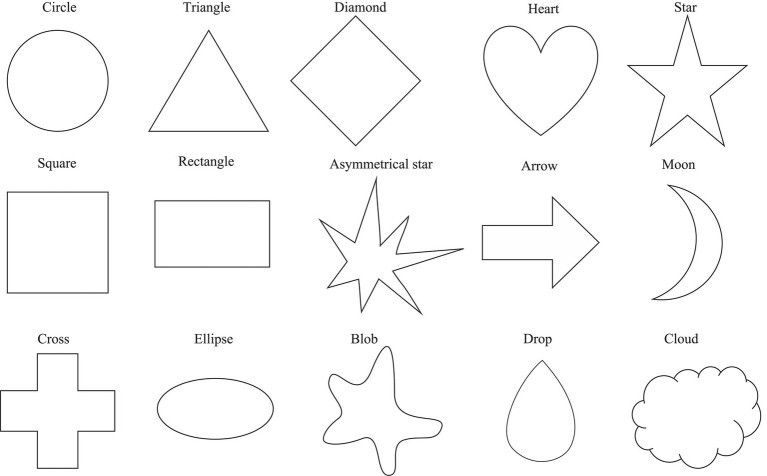
The 15 shape stimuli used in the study.

The 11 color patches and 15 geometric shapes were used as visual stimuli (Wan et al., 2014). The color patches included: Black (RGB: 0, 0, 0), Blue (RGB: 0, 0, 255), Brown (RGB: 165, 42, 42), Green (RGB: 0, 255, 0), Gray (RGB: 128, 128, 128), Orange (RGB: 255, 165, 0), Pink (RGB: 255, 192, 203), Purple (RGB: 128, 0, 128), Red (RGB: 255, 0, 0), White (RGB: 255, 255, 255), and Yellow (RGB: 255, 255, 0). The geometric shape stimuli were created using the Adobe Illustrator CC program (Adobe Systems Incorporated-2021). They were drawn in black ink in 1pt. line width, including Arrow, Asymmetrical star, Blob, Circle, Cloud, Cross, Diamond, Drop, Ellipse, Heart, Moon, Rectangle, Square, Star, and Triangle (as shown in Figure 1). In the first session of color–taste associations, the color stimulus was presented at the center of the screen in 190×190pixels. In the second session of the shape–taste associations, the shape stimulus was fitted in a 150×150-pixel-box. In the third session of the shape–color associations, the shape stimuli were the same as in the second session, and the color patches as choices were listed vertically in random in 38×38pixels.

### Design and Procedure

In this study, the participants undertook four sequential sessions (color–taste matching task, shape–taste matching task, shape–color matching task, and AQ-10 survey session). In addition, questionnaires on preference ratings, taste expectation, taste perception, and food behavior from other studies were simultaneously conducted. At the beginning of the online questionnaire survey, participants read the instructions and gave the consent to participate in it. After participants agreed to join by clicking the “agree” button, they were asked to fill in their demographic information, including age range, gender, diagnosed mental disorder, and birthplace. Participants were, then, prepared for the questionnaire survey. During the first session of the color-taste matching task, a color patch was presented at the center of the screen, and participants were asked to choose a taste that best matched the color of the five vertically listed basic taste words (i.e., sweet, sour, salty, umami, and bitter), by clicking on the taste word. Participants were directed to the Wikipedia website for an explanation of the term “Umami.” After making the choice, participants were asked how confident they were on their preceding match, indicated on a five-point horizontal scale with the following options, arranged from left to right: very unconfident (I am not sure and have chosen completely randomly), unconfident, neutral, confident, and very confident (I am very sure and confident about this match). The procedure was identical for the three matching tasks (i.e., color–taste, shape–taste, and shape–color). The color and shape targets were presented in the same order for all participants, and the choices were presented in random order. The shape targets in the shape–taste and shape–color matching tasks were presented in different orders.

**Figure 2:**
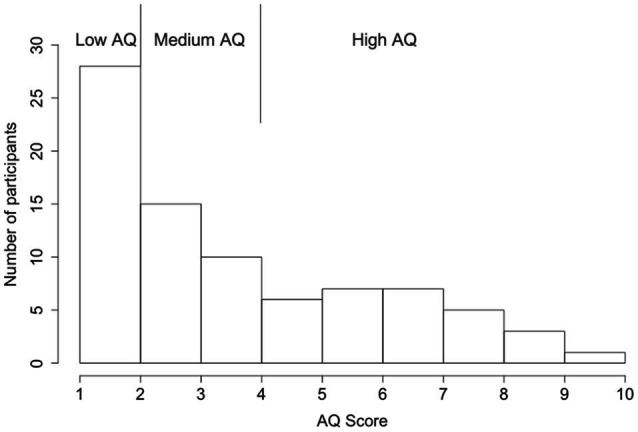
Distribution of participants’ AQ scores.

Finally, the participants completed a Japanese version of the AQ-10 questionnaire survey (Kurita et al., 2005; Maeda et al., 2017). The AQ-10 is a short version of the self-reported AQ-50 questionnaire that was developed to meet the need for a brief and sensitive screen for ASD (Allison et al., 2012; Booth et al., 2013). Participants were asked to rate the degree to which the content of each item fits them on a four-point Likert scale (“definitely agree,” “slightly agree,” “slightly disagree,” and “definitely disagree”). For example, item 1 states, “I prefer to do things with others rather than on my own.” The AQ was scored using a binary Likert scoring method (1-1-0-0; Baron-Cohen et al., 2001), that items that participants indicated either definite or slightly agreement with an autism trait were given a score of 1 and otherwise 0. A total AQ score was calculated by summing all scores for each item (1–10). Higher AQ scores indicate a greater magnitude of ASD traits. The sample of participants with the AQ score distribution is shown in Figure 2. To further understand the effect of autistic traits, participants were divided into three groups. While a score over 6 is considered the cut-off for a suspicion of ASD (National Institute for Health and Clinical Excellence, 2012; Booth et al., 2013; Murray et al., 2017), to balance the sample size across the three AQ groups, we used an AQ score of 5 as the criterion for the high AQ group (AQ≥5; 29 participants), an AQ score of 2 as the criterion for the low AQ group (AQ≤2; 28 participants), and participants with an AQ score between them were grouped into the medium AQ group (AQ=3, 4; 25 participants).

### Data Analysis

At first, the frequency of taste choice for each color (color–taste associations), the frequency of taste choice for each shape (shape–taste associations), and the frequency of color choice for each shape (shape–color associations) were calculated, respectively. A Chi-square test for independence was conducted to assess whether some choices were chosen more frequently than the others for certain targets. Moreover, adjusted residual analysis was used to show which choice was significantly associated with a certain target (i.e., the consensual color–taste/shape–taste/shape–color associations). Then, the proportion of making the consensual choices (i.e., significant cross-modal associations) in each participant was calculated. For example, if some participant chooses all the significantly associated tastes for each color, the proportion of the consensual color–taste associations should be 1. The AQ scores were not normally distributed (*W*=0.90, *p*<0.001; Shapiro–Wilk’s test); thus, log-transformed AQ scores were used in the analysis (Alan, 2010). Correlation analyses were performed between the proportions of consensual cross-modal associations and the autistic traits (i.e., log-transformed AQ scores; Pearson’s method; Hidaka and Yaguchi, 2018). Further, one-way Analysis of Variance (ANOVA) was used to examine the differences between the three AQ groups (i.e., high/medium/low AQ group) on the proportion of the consensual cross-modal associations in order to clarify overall trends. Data analyses were performed with R 4.0.0 software (R Core Team, 2020).

## Results

### Color–Taste Associations

**Figure 3:**
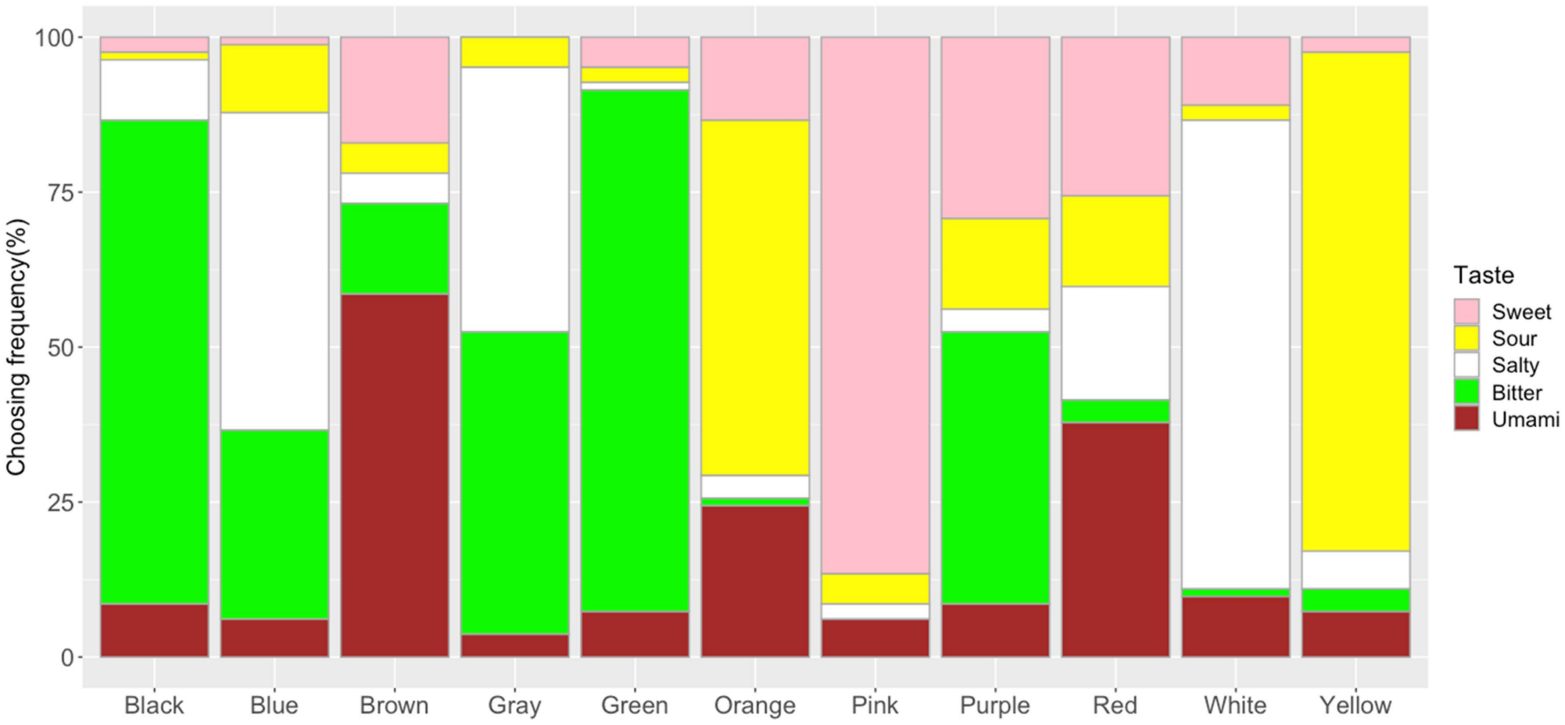
Frequency of taste choices for each color. Note that the frequency of the sweet, sour, salty, bitter, and umami taste terms for the color stimuli are represented by the fill-in colors pink, yellow, white, green, and brown, respectively.

The frequency of the taste choices for each color is shown in Figure 3 (see Supplementary Table S1 for more details). The Chi-square test for independence showed significant associations between colors and tastes (*x*^2^=1298.7, *df*=40, *p*<0.001, Cramer’s *V*=0.60). The adjusted residual analysis showed which taste was chosen more frequently than expected by chance for each color (a positive residual value indicates more frequently chosen than expected and a negative residual indicates less frequently chosen than expected; here, only positive residuals were used; Agresti, 2007; Chen et al., 2015a). Significant associations were observed between Pink and Sweet (*z*=17.19, *p*<0.001; Bonferroni corrected *z*>3.32, *p*<0.05; to test all 55 cells in the contingency table, the new alpha level is *α*=0.05/55=0.00091, with the corresponding critical value *z*=*N*(0,1)_1- α/2_=3.32); Yellow and Sour (*z*=15.41); Orange and Sour (*z*=9.69); Blue and Salty (*z*=7.43); White and Salty (*z*=13.23); Green and Bitter (*z*=11.82); Black and Bitter (*z*=10.53); Brown and Umami (*z*=10.92); Red and Umami (*z*=5.57); Gray and Salty (*z*=5.40); Gray and Bitter (*z*=4.35); Purple and Bitter (*z*=3.32).

The averaged confidence ratings for each of the observed color–taste associations were calculated. A significant correlation was observed between the choice frequency and the confidence rating of each color–taste association (*r*=0.28, *p*=0.04). Thus, the participants were more confident when making the more consensual color–taste associations.

#### Color–Taste Associations and AQ Scores

**Figure 4:**
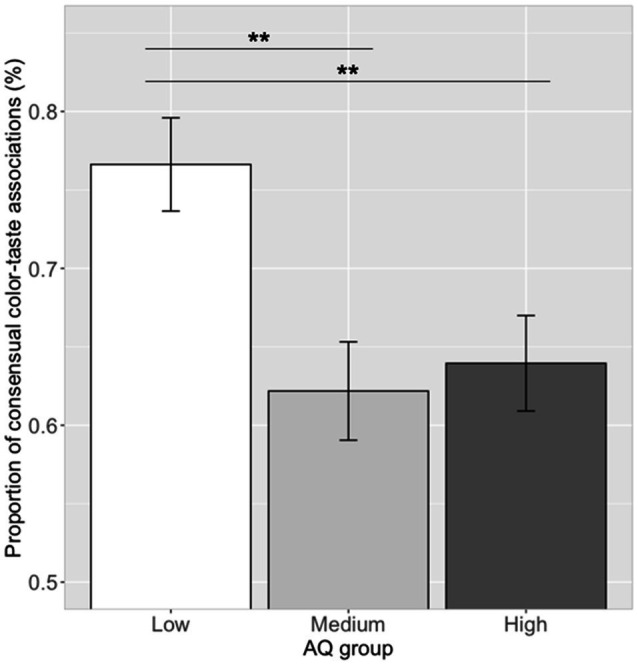
Proportion of consensual color–taste associations in the three AQ groups. Error bar represents the standard mean error. Asterisks indicate significant differences (**p<0.01).

The proportion of making the consensual color–taste associations (i.e., 12 significant color–taste associations) in each participant was calculated. Correlation analysis showed that the proportion of consensual color–taste associations and log (AQ) scores was significantly correlated (*r*=−0.24, *p*=0.027). Participants with lower AQ scores chose the more of the consensual color–taste associations. One-way ANOVA showed that the proportion of choosing consensual color–taste associations were significantly different among the three AQ groups [see Figure 4; *F*(2, 79)=6.68, *p*=0.002, η_p_^2^=0.14]. A *post hoc* pairwise comparison showed that the proportion of consensual color–taste associations in the low AQ group (mean=0.77, SD=0.16) was higher than those in the medium AQ group (mean=0.62, SD=0.16; Tukey’s HSD, *p*=0.003), and the high AQ group (mean=0.64, SD=0.16; Tukey’s HSD, *p*=0.008), and no difference between the medium and high AQ groups (Tukey’s HSD, *p*=0.91). Thus, participants in the low AQ group chose more of the consensual color–taste associations than those in the medium and high AQ groups.

### Shape–Taste Associations

**Figure 5:**
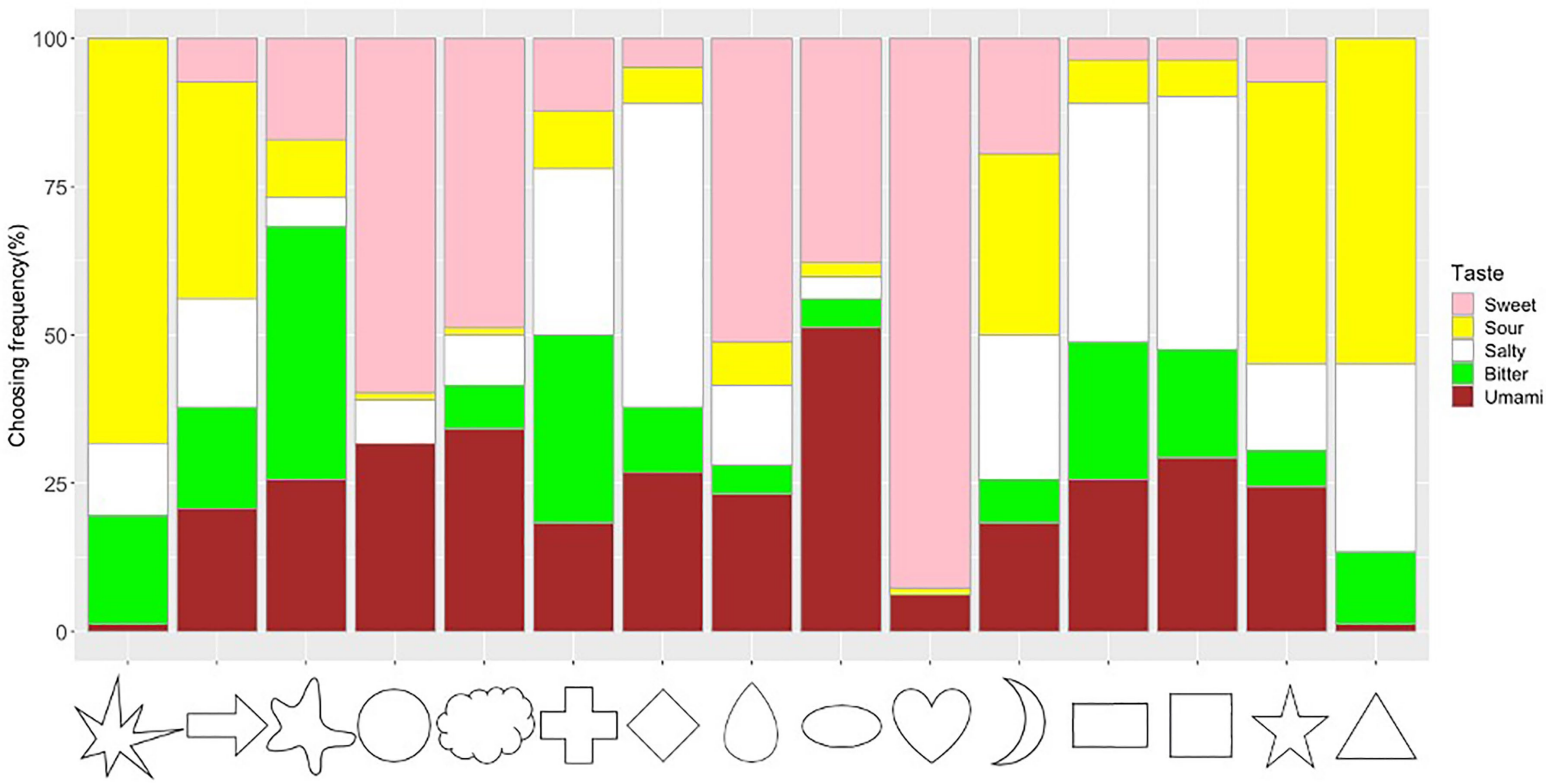
Frequency of taste choices for each shape. Note that the frequency of the sweet, sour, salty, bitter, and umami taste terms for the shape stimuli are represented by the fill-in colors pink, yellow, white, green, and brown, respectively.

The frequency of taste choices for each shape was calculated (Figure 5; Supplementary Table S2). The Chi-square test for independence showed that shapes and tastes were significantly associated (*x*^2^=998.84, *df*=56, *p*<0.001, Cramer’s V=0.45). The adjusted residual analysis showed that some tastes were chosen more frequently for specific shapes. For example, shapes with sharp angles were significantly associated with sour taste (e.g., Triangle: *z*=8.43; Star: *z*=6.69; Asymmetrical Star: *z*=11.61; Arrow: z=4.09; Bonferroni corrected *z*>3.40, *p*<0.05; to test all 75 cells in the contingency table, the new alpha level is α=0.05/75=0.00067, with the corresponding critical value *z*=*N*(0,1)_1-α/2_=3.40); round shapes were significantly associated with sweet taste (Heart: *z*=14.91, Circle: *z*=7.72, Cloud: *z*=5.32, Drop: *z*=5.86); right-angled shapes were significantly associated with salty taste (Rectangle: *z*=4.72, Diamond: *z*=7.29, Square: *z*=5.29). Other significant associations were observed between Ellipse and Umami (*z*=6.44), Blob and Bitter (*z*=7.92), and Cross and Bitter (*z*=4.93).

The averaged confidence ratings for each of the observed shape–taste associations were calculated. A significant correlation was observed between the choice frequency and the confidence ratings of each shape–taste association (*r*=0.29, *p*=0.015). Thus, participants were more confident when choosing the more consensual shape–taste associations.

#### Shape–Taste Associations and AQ Scores

**Figure 6:**
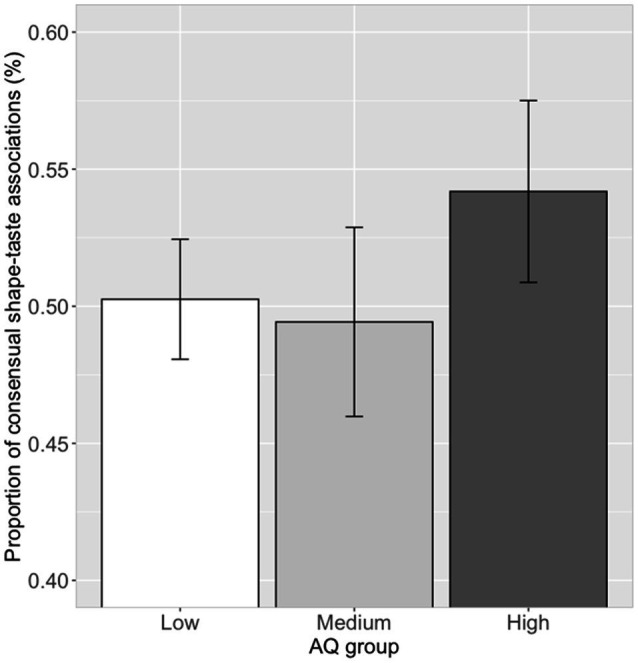
Proportion of consensual shape–taste associations in the three AQ groups. Error bar represents the standard mean error.

The proportion of choosing significant shape–taste associations (14 in all) were calculated for each participant. Correlation analysis showed that there was no significant correlation (*r*=0.07, *p*=0.54) between the proportion of consensual shape–taste associations and log (AQ) scores. Further ANOVA analysis showed no significant difference between the three AQ groups in the proportion of consensual shape–taste associations, *F*(2, 79)=0.72, *p*=0.49, η_p_^2^=0.02 (low AQ group: mean=0.50, SD=0.12; medium AQ group: mean=0.49, SD=0.17; high AQ group: mean=0.54, SD=0.18; Figure 6). Thus, autistic traits have little effect on the shape–taste associations.

### Shape–Color Associations

**Figure 7:**
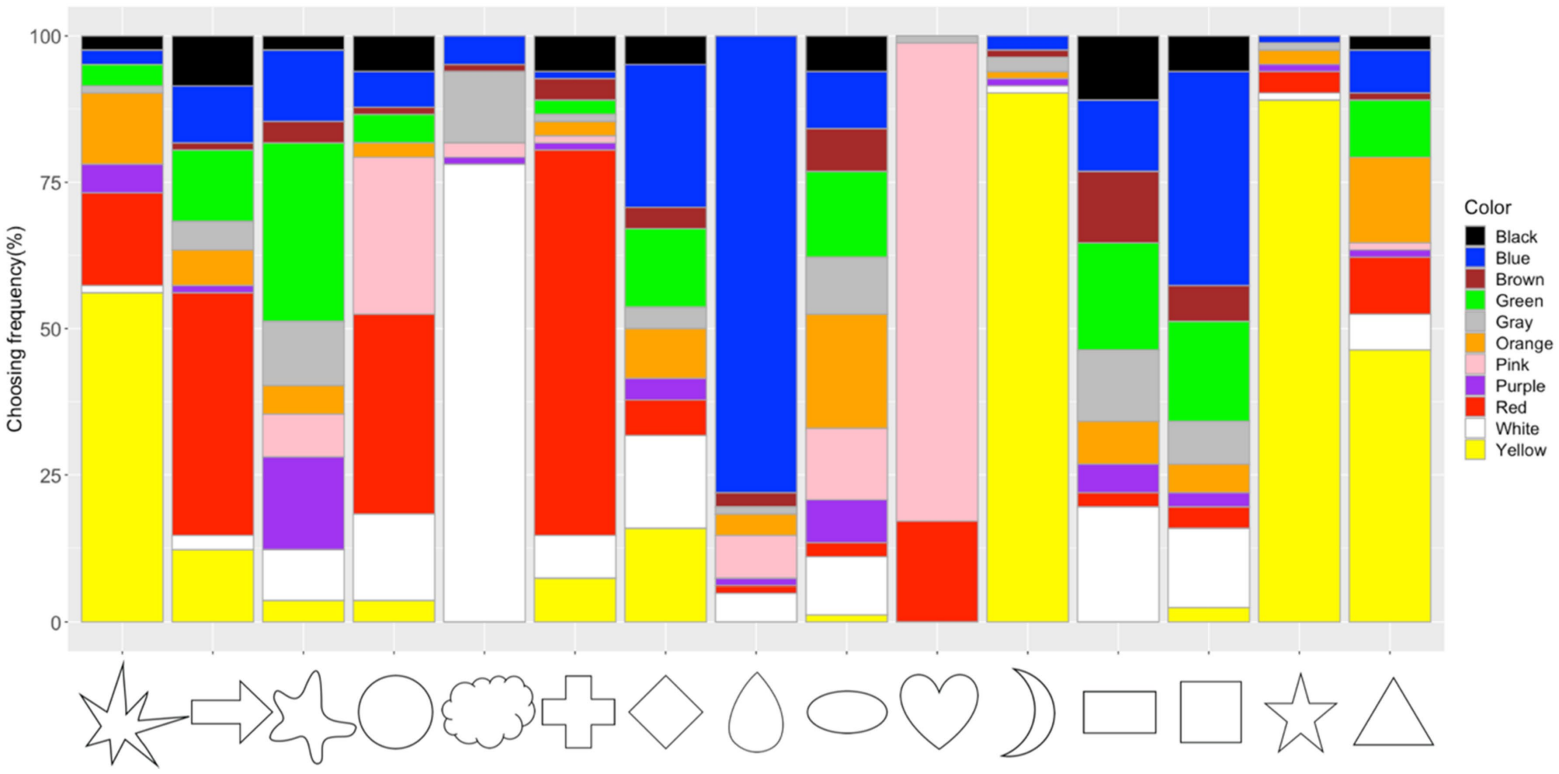
Frequency of color choices for each shape.

The frequency of color choices for each shape was calculated (Figure 7; Supplementary Table S3). The Chi-square test showed that there were significant associations between shapes and colors (*x*^2^=2468.9, *df*=140, *p*<0.001, Cramer’s *V*=0.45). The adjusted residual analysis showed that some colors were chosen more frequently for specific shapes [Bonferroni corrected *z*>3.61, *p*<0.05; to test all 165 cells in the contingency table, the new alpha level is α=0.05/165=0.00030, with the corresponding critical value *z*=*N*(0, 1)_1-α/2_=3.61]. The results showed that a circle was associated with red (*z*=5.63) and pink (*z*=5.63), a triangle with yellow (*z*=5.55), and a square with blue (*z*=6.15). A stereotypical pattern of shape–color associations was observed, in which people chose the color for a shape based on associative learning with co-occurrence in the environment. For example, pink was chosen more frequently for the heart shape (*z*=23.30), yellow was chosen more frequently for star (*z*=15.23), moon (*z*=15.50), and asymmetrical star (*z*=7.76) shapes, white for a cloud (*z*=18.79), blue for a drop (*z*=17.38), red for a cross (*z*=14.30), and so on. Other significant associations were observed between arrow with red (*z*=7.63), blob with green (*z*=7.42) and purple (*z*=6.78), ellipse and orange (*z*=5.32), and rectangle and brown (*z*=5.04). In order to restrain the observed significant shape–color associations, more Chi-square tests for goodness of fit and residual analysis were used on each shape to further test whether the color choices were still significant. Finally, the blob-purple (*z’*=1.77) and rectangle-brown (*z’*=0.31) associations failed to reach significance and were excluded from further analysis.

The averaged confidence ratings for each of the observed shape–color association were calculated. A significant correlation was observed between the choosing frequency and the confidence ratings of each shape–color association (*r*=0.39, *p*<0.001). Thus, participants were more confident when choosing the more consensual shape–color associations.

#### Shape–Color Associations and AQ Scores

**Figure 8:**
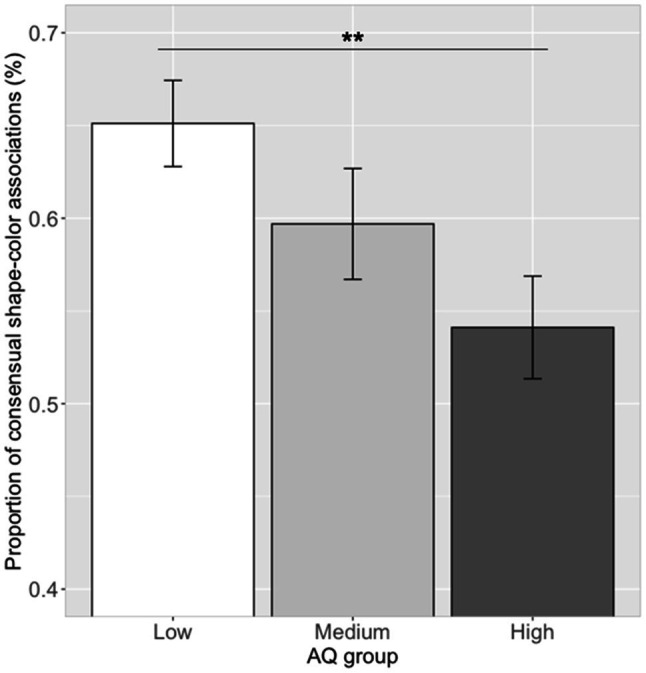
Proportion of consensual shape–color associations in the three groups. Error bar represents the standard mean error. Asterisks indicate significant differences (**p<0.01).

Correlation analysis between the proportion of the 14 significant shape–color associations and the log (AQ) scores showed a significant correlation (*r*=−0.29, *p*=0.007). Further ANOVA analysis showed that there was a significant difference in the proportion of choosing consensual shape–color associations between the three AQ groups [see Figure 8; *F*(2, 79)=4.35, *p*=0.016, η_p_^2^=0.10]. A *post hoc* pairwise comparison showed that the proportion of participants choosing consensual shape–color associations in the low AQ group (mean=0.65, SD=0.13) was higher than that of the high AQ group (mean=0.54, SD=0.15; Tukey’s HSD, *p*=0.009), and similar to that of the medium AQ group (mean=0.59, SD=0.15; Tukey’s HSD, *p*=0.34). Moreover, there was no significant difference between the high and medium AQ groups (Tukey’s HSD, *p*=0.31).

## Discussion

In the present study, we investigated the color–taste/shape–taste/shape–color associations in Japanese participants and the relationship between these associations and the autistic traits. The results showed that the participants established strong color–taste/shape–taste/shape–color associations (e.g., yellow–sour, triangle–sour, and triangle–yellow), which are consistent with previous findings (Wan et al., 2014; Chen et al., 2015a; Spence et al., 2015; Velasco et al., 2015, 2016a; Spence, 2019). Moreover, significant associations were observed between the proportion of choosing consensual color–taste/shape–color associations and autistic traits. That is, the participants with higher AQ scores chose fewer of the consensual color–taste/shape–color associations, while there was no difference in shape–taste associations. Thus, the autistic traits might play a role in the construction of color–taste/shape–color associations.

When forced to choose a taste matching a color, our participants showed specific patterns of color–taste associations. For example, pink color was associated with sweet, yellow and orange colors were associated with sour, green with bitter, blue with salty, brown and red with umami. For non-chromatic colors, white was associated with salty, black with bitter, and gray with both salty and bitter tastes. These results were consistent with previous findings, suggesting associations between red/pink with sweet, yellow with sour, white/blue with salty, black/green with bitter among different culture backgrounds (Déribéré, 1978; O’Mahony, 1983; Koch and Koch, 2003; Skrandies and Reuther, 2008; Tomasik-Krótki and Strojny, 2008; Wan et al., 2014; Spence et al., 2015; Saluja and Stevenson, 2018; Spence and Levitan, 2021). These color–taste mapping may come from statistical learning with colors of foods/drinks in the world (e.g., pink pairing with sweet candy, green vegetables tasting bitter, and yellow lemons tasting sour; Koch and Koch, 2003; Spence et al., 2015; Saluja and Stevenson, 2018; Higgins and Hayes, 2019). Culturally specific color–taste associations were also observed; for example, Japanese people associated brown/red with umami tastes, while no specific color–umami association was observed among four cultural backgrounds in a previous study (Tomasik-Krótki and Strojny, 2008; Wan et al., 2014; Saluja and Stevenson, 2018). This might be related to the fact Japanese people are more familiar with the umami taste and thus, the taste is more important due to its significant involvement in Japanese daily food behavior (Kawamura and Kare, 1987; Lindemann et al., 2002; Lioe et al., 2010). It is well-known that the umami taste is first identified by a Japanese chemist – Ikeda Kikunae in 1909, referring to the savory taste from meat or seaweed. Ikeda also suggested that umami taste would be associated with yellow and red colors (Ikeda, 2002). Here, the observed brown/red-umami associations might be stemmed from the Japanese seasoning sources (e.g., “Miso,” “soy source,” and so on) which are mainly colored in brown and red (Otsuka, 1998; Lioe et al., 2010; Hajeb and Jinap, 2015). Compared with a previous study using the same color stimuli (Wan et al., 2014), robust color–taste associations were observed in Japanese people (see Figure 3). Around 80% of the participants chose sweet taste for pink, sour for yellow, bitter for green, salty for white, and bitter for black, suggesting that our participants were highly consistent with the taste choices for some colors. It should be noted that most of the participants were from east Japan and thus, might share a highly similar regional food behavior. These results provide further evidence for the correspondence hypothesis between colors and tastes (Koch and Koch, 2003; Spence et al., 2015; Higgins and Hayes, 2019; Spence, 2019; Spence and Levitan, 2021).

Specific shape–taste associations were also observed. For example, sharp shapes were associated with sour (e.g., asymmetrical star, star, triangle, and arrow), rounded shapes were associated with sweet (e.g., heart, circle, drop, and cloud) and umami (i.e., ellipse), and right-angled shapes were associated with salty (e.g., diamond, rectangle, and square). These results showed both consistent and culturally specific shape–taste associations [e.g., associations between cross/rectangle/square with bitter, and cloud/heart/star with sweet in Wan et al. (2014); round shapes associate with sweet tastes, angular shapes associate with sour/bitter/salty taste in Spence and Ngo (2012) and Velasco et al. (2015, 2016b)]. Specifically, Japanese people showed umami–ellipse and right-angled shape–salty associations. Similarly, as the umami taste is more familiar to Japanese people, it allows them to establish specific associations with both colors and shapes. When mapped onto colors, both umami and sweet were associated with red (umami–red/brown and sweet–red/pink). For shape–taste associations, both umami and sweet were associated with rounded shapes (umami–ellipse and sweet–circle/heart/drop/cloud). This might be related to the similarity between umami and sweet taste (e.g., familiarity and pleasantness; Obrist et al., 2014; Spence and Levitan, 2021), and/or that their sensory receptors share some common subunit (Li et al., 2002). Obrist et al. (2014) described both umami and sweet taste as full, mouth-filling experience, and umami also tasted as with lots of things to it, which may lead to the umami–ellipse association. Several studies proposed emotional/semantic correspondence hypotheses to explain shape–taste associations (Spence and Deroy, 2012b, 2013a; Salgado-Montejo et al., 2015; Velasco et al., 2015, 2016b,c; Blazhenkova and Kumar, 2018; Turoman et al., 2018; De Sousa et al., 2020; Hamamoto et al., 2020; Motoki and Velasco, 2021). For example, Velasco et al. (2016b) suggested that semantic meanings (e.g., hedonic) underlying shapes and tastes could explain some of the shape–taste associations. Turoman et al. (2018) showed that the affective factors of pleasantness and threat underlie some shape–taste correspondences. The observed shape–taste associations might stem from a common semantic basis. For instance, both the sharp shapes and sour taste may convey some exciting/stimulating semantic meanings, and the round shapes and sweet taste may share hedonic/pleasant semantic information, leading to these shape–taste associations.

For the shape–color associations, we observed that our participants tended to associate shapes with colors more frequently encountered in the environment. For example, moon and star shapes were associated with yellow, heart with pink, cloud with white, drop with blue, and cross/arrow with red. The heart–pink association (instead of heart–red) might be related to the Japanese cultural background, that Japanese people often use pink–heart related symbols to express “Kawaii (lovely/cute)” emotion/semantic meanings. Thus, the co-occurrences and regularities in the world might lead to some of the observed shape–color associations (Jacobsen and Wolsdorff, 2007; Chen et al., 2015a; Hanada, 2019). Moreover, for some geometric shapes, such as circle associated with red and pink, triangle with yellow, and square with blue, these associations were consistent with previous findings, and some might be explained by semantic associations (e.g., “warm/cold”; Albertazzi et al., 2013; Chen et al., 2015a, 2016). Strictly speaking, shape–color associations are intramodal, mostly studied within visual dimensions, and grounded in the field of crossmodal correspondence (Dreksler and Spence, 2019). Colors and shapes are commonly used in crossmodal correspondences, while the associations between colors and shapes have been relatively less studied. Learning shape–color associations can provide further evidence for the connections between sensory dimensions. Future studies may examine the relationship between intramodal and crossmodal correspondences to better understanding the binding of sensory information.

The ability to establish specific crossmodal correspondences between sensory dimensions could be influenced by individual differences (Bremner et al., 2013; Fernández-Prieto et al., 2015; Hamilton-Fletcher et al., 2018). Here, we observed that our participants with higher AQ scores tended to make fewer of the consensual color–taste/shape–color associations, suggesting that the autistic traits play a role in constructing crossmodal correspondences. This result was consistent with previous findings that individuals with autism showed atypical sensory processing and deficits in binding sensory information across modalities (Oberman and Ramachandran, 2008; Foss-Feig et al., 2010; Cascio et al., 2012a; Kujala et al., 2013; Occelli et al., 2013; Visser et al., 2013; Stevenson et al., 2014b, 2016; Wada et al., 2014; Gold and Segal, 2017; Król and Ferenc, 2020; Yaguchi and Hidaka, 2020), as evident in audio–visual processing (Gelder et al., 1991; Mongillo et al., 2008; Irwin et al., 2011; Collignon et al., 2013; Bebko et al., 2014; Stevenson et al., 2014b; Hidaka and Yaguchi, 2018), audio–tactile processing (Russo et al., 2010), and visual–tactile processing (Cascio et al., 2012a; Noel et al., 2020). For example, Occelli et al. (2013) found that children with ASD showed lower proportion of expected takete–maluma associations, and the performance varied as a function of the severity of the symptomatology. Hidaka and Yaguchi (2018) showed that the brightness–loudness association and a newly learned association (motion direction–pitch pair) correlated with autistic traits, suggesting that ASD traits are involved in sensory associative learning. According to the Bayesian priors (“hypo-priors”) hypothesis, the sensory-perceptual representations of the world are built based on a Bayesian statistic; based on prior knowledge, a weighted, generated model is constructed from sensory inputs and an internal probability map. Individuals with autism tend to have a decreased ability to perform statistical learning with co-occurrences and regularities in the environment, leading to difficulties in associating and integrating sensory information across modalities (Pellicano and Burr, 2012; Stevenson et al., 2014a, 2017). The autistic effect was observed on color–taste/shape–color associations, which might be mainly constructed through statistical learning with regularities in the environment (Koch and Koch, 2003; Spence et al., 2015; Saluja and Stevenson, 2018; Hanada, 2019; Higgins and Hayes, 2019; Spence, 2019), while no significant autistic effect was observed on shape–taste associations, which might be mainly explained by semantic meaning correspondences (e.g., hedonic dimensions; Salgado-Montejo et al., 2015; Velasco et al., 2015, 2016b,c; Blazhenkova and Kumar, 2018; Turoman et al., 2018; De Sousa et al., 2020; Hamamoto et al., 2020; Motoki and Velasco, 2021). Some studies suggested that the semantic/pleasant ratings on sensory inputs are similar between individuals with autism and controls (Cascio et al., 2012b; Galle et al., 2013; Damiano et al., 2014; Singh, 2019). For example, Damiano et al. (2014) reported that individuals with ASD and neurotypical controls showed no differences in hedonic response to sweet taste. Moreover, shape–taste associations are likely different in nature from color–taste associations. Given flavors always have colors attached whereas flavors often do not have a shape, as in drinks that simply conform to the receptacle in which they are placed (Spence and Deroy, 2012b, 2013a). Besides, the consensual shape–taste associations (mean frequency=51.39%, SD=14.37) were relatively weaker than the consensual color–taste associations (mean frequency=62.09%, SD=17.82) and shape–color associations (mean frequency=55.31%, SD=22.91), which might influence the strength of relationship with autistic traits.

One limitation of the present study is that we used the Japanese Kanji word “旨味 (Umami)” to represent the “うま味 (Umami)” in the questionnaire. The word “うま味 (Umami)” is the technical word for the umami taste, while the “旨味 (Umami)” also indicates deliciousness. According to multiple Japanese dictionaries (e.g., Digital Daijisen and Wikipedia), both of them convey the same meaning of umami taste. Another limitation is that the four sessions (i.e., color–taste association, shape–taste association, shape–color association, and AQ survey) in the questionnaire were sequentially presented due to the questionnaire design of the platform. It may influence the choices that participants made. Future studies should present different sessions in a randomized order. In addition, we used the forced choice method for the questionnaire to meet the previous study (Wan et al., 2014). In some combinations, multiple associations could exist in some participants, and thus, using Check-All-That-Apply (CATA) method might be considered in future studies (Dooley et al., 2010).

For background of the limited food preference, not only the associations between taste (color/shape) and vision but also various factors, such as texture, sound, and smell, could be important. It would be interesting to investigate the relationship between autistic traits and other crossmodal correspondences, such as sound–taste association (Wang et al., 2016; Motoki et al., 2020), as well as their relationship to the limited food preference in ASD.

In summary, the present study showed specific and robust color–taste/shape–taste/shape–color associations in Japanese people with some culturally specific influences on the observed associations. Moreover, the degree of autistic traits was found to be related to the strength of color–taste/shape–color associations, but not to shape–taste associations. This might be explained by a Bayesian prior hypothesis that autistic perception may have different levels of statistical learning with regularities in the world, leading to a lower prior knowledge effect on constructing those learned associations. These results provide further evidence for the “hypo-priors” account for autistic perception and shed light on the nature of visual–taste associations. Future studies may work on crossmodal correspondences in diagnosed ASD patients, to further address the associative learning effect using novel crossmodal correspondences, and to better understand the autistic effect on such correspondences.

## Data Availability Statement

The datasets presented in this study can be found in online repositories. The names of the repository/repositories and accession number(s) can be found in the article/Supplementary Material.

## Ethics Statement

This study was reviewed and approved by the Ethics Committee of the National Rehabilitation Center for Persons With Disabilities (2020-082). This study was conducted online, participants agreed to join the study by clicking the “agree” button at the beginning of the online questionnaire survey, after they read the instruction of participating in the study. Participants under 18 joined the questionnaire with the agreement of their legal guardian/next of kin.

## Author Contributions

NC, KW, and MW designed and performed the experiments. NC analyzed the data and wrote the manuscript. KW and MW provided the critical revisions. All authors contributed to the article and approved the submitted version.

## Funding

This work was supported by the Grant-in-Aid for Scientific Research (grant numbers: 17H06344, 17H00753, 19K22885, 20H04595, 20K22296, 21K13759, and 21H05053) from the Japan Society for the Promotion of Science.

## Conflict of Interest

The authors declare that the research was conducted in the absence of any commercial or financial relationships that could be construed as a potential conflict of interest.

## Publisher’s Note

All claims expressed in this article are solely those of the authors and do not necessarily represent those of their affiliated organizations, or those of the publisher, the editors and the reviewers. Any product that may be evaluated in this article, or claim that may be made by its manufacturer, is not guaranteed or endorsed by the publisher.

## Footnotes

^1^ https://forms.gle/K3UjFcN7pVU6AFwi8
