# Supplementary material for: People With High Autistic Traits Show Fewer Consensual Crossmodal Correspondences Between Visual Features and Tastes
Source: Front Psychol. 2021 Sep 8;12:714277. doi: 10.3389/fpsyg.2021.714277 (PMC8457010; doi:10.3389/fpsyg.2021.714277)
Supplement: Supplementary file 2 [file Table_1.docx]

**Supplementary materials**

1. **Contingency tables**

Table S1. Choosing frequency of taste for each color (%)

|  | Black | Blue | Brown | Gray | Green | Orange | Pink | Purple | Red | White | Yellow |
| --- | --- | --- | --- | --- | --- | --- | --- | --- | --- | --- | --- |
| Sweet | 2.44 | 1.22 | 17.07 | 0.00 | 4.88 | 13.41 | ***86.59*** | 29.27 | 25.61 | 10.98 | 2.44 |
| Sour | 1.22 | 10.98 | 4.88 | 4.88 | 2.44 | ***57.32*** | 4.88 | 14.63 | 14.63 | 2.44 | ***80.49*** |
| Salty | 9.76 | ***51.22*** | 4.88 | ***42.68*** | 1.22 | 3.66 | 2.44 | 3.66 | 18.29 | ***75.61*** | 6.10 |
| Bitter | ***78.05*** | 30.49 | 14.63 | ***48.78*** | ***84.15*** | 1.22 | 0.00 | ***43.90*** | 3.66 | 1.22 | 3.66 |
| Umami | 8.54 | 6.10 | ***58.54*** | 3.66 | 7.32 | 24.39 | 6.10 | 8.54 | ***37.80*** | 9.76 | 7.32 |

*Note. Cells indicate significant color-taste associations are shown in bold (p < .05).*

Table S2. Choosing frequency of taste for each shape (%)

|  | Circle | Triangle | Square | Star | A-star | Heart | Blob | Cloud | Cross | Diamond | Drop | Moon | Ellipse | Rectangle | Arrow |
| --- | --- | --- | --- | --- | --- | --- | --- | --- | --- | --- | --- | --- | --- | --- | --- |
| Sweet | ***59.76*** | 0.00 | 3.66 | 7.32 | 0.00 | ***92.68*** | 17.07 | ***48.78*** | 12.20 | 4.88 | ***51.22*** | 19.51 | 37.80 | 3.66 | 7.32 |
| Sour | 1.22 | ***54.88*** | 6.10 | ***47.56*** | ***68.29*** | 1.22 | 9.76 | 1.22 | 9.76 | 6.10 | 7.32 | 30.49 | 2.44 | 7.32 | ***36.59*** |
| Salty | 7.32 | 31.71 | ***42.68*** | 14.63 | 12.20 | 0.00 | 4.88 | 8.54 | 28.05 | ***51.22*** | 13.41 | 24.39 | 3.66 | ***40.24*** | 18.29 |
| Bitter | 0.00 | 12.20 | 18.29 | 6.10 | 18.29 | 0.00 | ***42.68*** | 7.32 | ***31.71*** | 10.98 | 4.88 | 7.32 | 4.88 | 23.17 | 17.07 |
| Umami | 31.71 | 1.22 | 29.27 | 24.39 | 1.22 | 6.10 | 25.61 | 34.15 | 18.29 | 26.83 | 23.17 | 18.29 | ***51.22*** | 25.61 | 20.73 |

*Note. Cells indicate significant shape-taste associations are shown in bold (p < .05), A-star represents asymmetrical star.*

Table S3. Choosing frequency of color for each shape (%)

|  | Circle | Triangle | Square | Star | A-star | Heart | Blob | Cloud | Cross | Diamond | Drop | Moon | Ellipse | Rectangle | Arrow |
| --- | --- | --- | --- | --- | --- | --- | --- | --- | --- | --- | --- | --- | --- | --- | --- |
| Red | ***34.15*** | 9.76 | 3.66 | 3.66 | 15.85 | 17.07 | 0.00 | 0.00 | ***65.85*** | 6.10 | 1.22 | 0.00 | 2.44 | 2.44 | ***41.46*** |
| Pink | ***26.83*** | 1.22 | 0.00 | 0.00 | 0.00 | ***81.71*** | 7.32 | 2.44 | 1.22 | 0.00 | 7.32 | 0.00 | 12.20 | 0.00 | 0.00 |
| Orange | 2.44 | 14.63 | 4.88 | 2.44 | 12.20 | 0.00 | 4.88 | 0.00 | 2.44 | 8.54 | 3.66 | 1.22 | ***19.51*** | 7.32 | 6.10 |
| Yellow | 3.66 | ***46.34*** | 2.44 | ***89.02*** | ***56.10*** | 0.00 | 3.66 | 0.00 | 7.32 | 15.85 | 0.00 | ***90.24*** | 1.22 | 0.00 | 12.20 |
| Green | 4.88 | 9.76 | 17.07 | 0.00 | 3.66 | 0.00 | ***30.49*** | 0.00 | 2.44 | 13.41 | 0.00 | 0.00 | 14.63 | 18.29 | 12.20 |
| Purple | 0.00 | 1.22 | 2.44 | 1.22 | 4.88 | 0.00 | 15.85 | 1.22 | 1.22 | 3.66 | 1.22 | 1.22 | 7.32 | 4.88 | 1.22 |
| Blue | 6.10 | 7.32 | ***36.59*** | 1.22 | 2.44 | 0.00 | 12.20 | 4.88 | 1.22 | 24.39 | ***78.05*** | 2.44 | 9.76 | 12.20 | 9.76 |
| Brown | 1.22 | 1.22 | 6.10 | 0.00 | 0.00 | 0.00 | 3.66 | 1.22 | 3.66 | 3.66 | 2.44 | 1.22 | 7.32 | 12.20 | 1.22 |
| White | 14.63 | 6.10 | 13.41 | 1.22 | 1.22 | 0.00 | 8.54 | ***78.05*** | 7.32 | 15.85 | 4.88 | 1.22 | 9.76 | 19.51 | 2.44 |
| Gray | 0.00 | 0.00 | 7.32 | 1.22 | 1.22 | 1.22 | 10.98 | 12.20 | 1.22 | 3.66 | 1.22 | 2.44 | 9.76 | 12.20 | 4.88 |
| Black | 6.10 | 2.44 | 6.10 | 0.00 | 2.44 | 0.00 | 2.44 | 0.00 | 6.10 | 4.88 | 0.00 | 0.00 | 6.10 | 10.98 | 8.54 |

*Note. Cells indicate significant shape-color associations are shown in bold (p < .05). A-star represents asymmetrical star.*

1. **Questionnaire survey**

Welcome to the online questionnaire survey on relationship between colors, shapes, and basic tastes. This study aims to understand the food difficulties faced by people with developmental disabilities, to develop better solutions for solving the unbalanced food behavior problems.

The results of this survey will be published as conference presentations, academic papers, and databases, and may also be used as publicity and educational materials. Since this is an anonymous survey, please do not write anything related to personal information (name, address, etc.) in the free text field. For more details about this study, please refer to the link of "Instruction for Research Participants”. If you are under 20 years old, please obtain the consent of your parent or guardian before filling out this form.

Thank you very much for your time.

What is your age range?

○ 15~19

○ 20~29

○ 30~39

○ 40~49

○ 50~59

○ 60~

What is your gender?

○ Male

○ Female

○ Others/prefer not to answer

Have you ever been diagnosed with a developmental disability or other neuropsychiatric disorder? Please select all that apply (multiple answers are possible).

○ No diagnosis (generally neurotypical)

○ Autism spectrum disorders (autism/ Asberger syndrome/ pervasive developmental disorder)

○ Attention deficit hyperactivity disorder

○ Learning disabilities

○ Intellectual disability

○ Other neuropsychiatric disorders / disorders

○ Unknown (including undiagnosed / suspected)

If you selected "unknown (including undiagnosed or suspected)" for "other neuropsychiatric diseases/disorders" in the previous question, please provide the specific name of the diagnosis (including the judgment by the public institution).

_______________________________________

What is your hometown?

○ Hokkaido

○ Tohoku

○ Kanto

○ Chubu

○ Kinki

○ Chukoku

○ Shikoku

○ Kunsho/Okinawa

○ Outside of Japan

**Session 1: Relationship between colors and tastes**

In this session, the relationship between color and basic taste will be investigated. Please choose one taste that best matches the presented color.

*If you are confused about Umami taste, please refer to the following link.

<https://ja.wikipedia.org/wiki/%E3%81%86%E3%81%BE%E5%91%B3>

Please choose one taste that best matches with the color below:


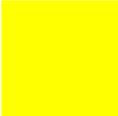


- Sweet
- Sour
- Salty
- Umami
- Bitter

Please indicate your confidence about this choice: 1 [Not at all, I chose it completely randomly]. 5 [Very much, it feels completely right].

Not at all ○1 ○2 ○3 ○4 ○5 very much

(*) A total of 11 colors are presented in this session, including: Yellow, Green, Red, Blue, Black, Brown, Pink, White, Orange, Purple, and Gray, presented in sequence.

**Session 2: Relationship between shapes and tastes**

In this session, the relationship between shape and basic taste will be investigated. Please choose one taste that best matches the shape.

Please choose one taste that best matches with the shape below:


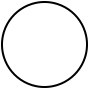


- Sweet
- Sour
- Salty
- Umami
- Bitter

Please indicate your confidence about this choice: 1 [Not at all, I chose it completely randomly]. 5 [Very much, it feels completely right].

Not at all ○1 ○2 ○3 ○4 ○5 very much

(*) A total of 15 shapes will be presented, including: circle, triangle, square, star, heart, cloud, asymmetrical star, diamond, ellipse, moon, cross, arrow, rectangle, drop, and blob, presented in sequence.

**Session 3: Relationship between color and shape**

In this session, the relationship between color and shape will be investigated. Please choose one color that best matched with the presented shape.

Please choose one color that best matches the shape below:


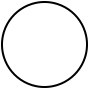


-
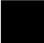

-
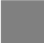

-
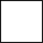

-
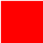

-
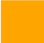

-
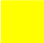

-
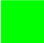

-
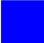

-
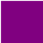

-
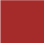

-
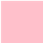


Please indicate your confidence about this choice: 1 [Not at all, I chose it completely randomly]. 5 [Very much, it feels completely right].

Not at all ○1 ○2 ○3 ○4 ○5 very much

(*) A total of 15 shapes will be presented, including: triangle, circle, square, star, heart, moon, cloud, cross, ellipse, diamond, arrow, drop, rectangle, blob, and asymmetrical star, presented in sequence.

**Last session: Personality traits**

For each statement below, choose one response that best describes how strongly that statement applies to you:

○Definitely Agree ○Slightly Agree ○Slightly Disagree ○Definitely Disagree

1. I prefer to do things with others rather than on my own.
2. Other people frequently tell me that what I’ve said is impolite, even though I think it is polite.
3. I tend to have very strong interests, which I get upset about if I can’t pursue.
4. When I’m reading a story, I find it difficult to work out the characters’ intentions.
5. I would rather go to the theater than to a museum.
6. I am often the last to understand the point of a joke.
7. I find it easy to work out what someone is thinking or feeling just by looking at their face.
8. I like to collect information about categories of things (e.g., types of cars, birds, trains, plants).
9. I find it difficult to imagine what it would be like to be someone else.
10. I find it difficult to work out people’s intentions.
